# Gazing Long into a Clinical and Social Abyss? Treating Hypertrophic Scarring and Keloids

**DOI:** 10.1089/rej.2021.0037

**Published:** 2021-08-16

**Authors:** Alison R. Carter, Benjamin M. Davies, David A. Brindley

**Affiliations:** Biolacuna Ltd, Wantage, United Kingdom.

**Keywords:** hypertrophic scars, keloids, dermatology, drug development, regenerative medicine

## Abstract

This commentary discusses the unmet clinical and social needs associated with hypertrophic scars and keloids. The authors critically appraise these issues within the context of contemporary clinical standards of care and social mores catalyzed by the COVID-19 pandemic.

“Do everything like someone is gazing at you.”

—Epicurus

Before the COVID-19 pandemic began to extend its grip across our everyday lives, various sections of society had begun to observe the challenges of an increased gaze into people's day-to-day existence, whether caused through the increased popularity of, and trend for, social media accounts, tracking capabilities through modern technology (telephones, computing devices, CCTV—including facial recognition), traditional media scrutiny grown more instant than ever through the internet, or other means. This took a somewhat unexpected turn during national lockdowns—formalizing remote working has in some organizations gradually led to an increased gaze into employees' calendars, with the natural endpoint for some being the charting and accounting of every minute of the working day. More commonly, the pandemic has led to a mushrooming of calls through videoconferencing, which, in turn, has led to colleagues sitting, sometimes for several hours each day, scrutinizing intensely rows of boxes showing their peers' faces with an array of home or virtual backdrops.

This has led to some comic newspaper headlines: for instance, people appearing upside down on camera or, (in)famously, during legal proceedings through a cat filter.^1^ However, there have also been a number of reports of the physical and emotional (dis)stresses prompted by the sometimes intense nature of back-to-back video calls, and of the stress caused to some by the intrusion into what used to be, and will hopefully once again become, a private space—the home.^2^ The lines between work and homelife have become blurred.^3^

In addition to the above, the gaze of other people can be bruising in other ways. The early stages of the pandemic saw a clamor for procedures to change people's looks while shaded by their face coverings: whether rooted in physical or emotional health needs, cosmetic surgery and orthodontistry saw stiff trade where possible within pandemic restrictions.^4^ Prepandemic, the rise of social media had also seen an increase in concerns over the potential damage caused by a drive to look “perfect”—however perfection may be defined in each culture, society, and generation.

And yet, despite centuries of research and millennia of dreaming, there remains only one way to appear “perfect”: by using software filters and technical wizardry.

Skin “perfection” is widely held up as a goal to be attained, and is a quest that affects both old and young, and those who have suffered injury or none. During prolonged lockdowns and inspired by social factors including the above and their research interests, the authors have considered an offshoot of this area—the treatment of hypertrophic scars and keloids and their effectiveness—to see what new insights can be gained in this area.

To explain a little further, hypertrophic scars are described as those that are raised beyond the skin level, but retained within the boundaries of the original scar, whereas keloids extend beyond the original scar boundary. Both are a significant cause of morbidity and psychological distress to patients.

This focus is timely because, as alluded to above, an unexpected consequence of the pandemic has been an increase in the percentage of our daily lives spent on show, framed in “close-up,” which for some with dermatological conditions has contributed to additional stress and a feeling of helpless exposure: psychoemotional stress.^5,6^ For those affected by dermatological conditions (e.g., keloid or acne scars, cold sores) that adversely impact their physical appearance—whether transiently or permanently—there are multiple evils stemming from what, from their perspective, may have become the “overexposure” of videoconferencing. First, there is the matter of feeling obliged to display publicly, and potentially for prolonged amounts of time, something about their body over which they may feel self-conscious. This can lead to psychological distress and, in some cases, trauma.^7^ Second, and perhaps more cruelly for those patients impacted, the emotional stress that enforced public exposure creates can augment the extent and frequency of some skin scars, including keloids.^8^

Let us pause a moment to note that keloids and other scars can impact different ethnic groups differently (affecting 16% of the population in Zaire, but only 0.09% of the population in the United Kingdom).^9–11^ This is, therefore, a condition, or range of conditions, affecting those who frequently have less access to corrective treatments. However, that said, and implicit in the above, few are impervious to the harsh glare of society when it comes to physical appearance, which, sometimes in combination with underlying currents of the ageism and sexism “revealed” annually in studies across many cultures, drives sales of over-the-counter cosmetic products with relatively little demonstrated efficacy at sometimes dizzying price points. This is something that affects us all.

The efforts of the medical and research community to overcome the problems associated with hypertrophic scars and keloids have led to the development of a wide variety of treatments, including those listed in Table 1. To put this into perspective, the treatment of skin scarring already represented a 12 billion U.S. dollar market by 2009.

**Table 1.** Modalities Employed in the Treatment of Hypertrophic Scars and Keloids

Invasive	Noninvasive	
	Pharmacological	Physical
Surgical excision Brachytherapy Radiotherapy Laser treatment Intralesional corticosteroids Intralesional 5-fluorouracil Intralesional bleomycin Botulinum toxin	Topical corticosteroids Onion extract	Silicone gel sheets Silicone gel spray Pressure therapy Massage

This array of treatments has been utilized in burn scars, hypertrophic scarring, and keloids. Although studies have demonstrated a degree of success in different situations, the fact that such a broad selection of treatments is being utilized and that there is no consensus on their use highlights the difficulty in establishing the effectiveness of these treatments. This is exacerbated by the multiplicity of methods used to establish effectiveness—from physical measurements of scars to purely patient-reported outcomes.

If the reader shares a mindset similar to the authors, they will perhaps be intrigued by the disparate avenues explored and appearance of potential treatments such as onion extract. Our study (to be published separately and providing details beyond the scope of this editorial) explores in greater depth the diverse treatments that have been used. None of these treatments have become widely established as a standard of care, a fact that in itself indicates that none have yet been shown to have the efficacy versus cost/risk profile that would support widespread adoption.

Most intervention types have been tried across different types of scarring (e.g., burn vs. nonburn) and populations. In developing a new treatment, it would be important to determine whether the treatment is suitable for all populations affected by the condition. A new product would also need to demonstrate an appropriate level of effectiveness. Intralesional steroids are, for example, used as a first-line treatment for keloids and hypertrophic scars. Their continued clinical use contrasts a recent meta-analysis that concluded that intralesional steroid use in hypertrophic scars and keloids was no better than therapies such as with 5-fluorouracil in the medium to long term, but was associated with more complications.^12^

The limitations of all currently available treatments are, perhaps, the single common and unifying phenomenon that has prevented any one treatment from becoming widely adopted as a standard of care. Although intralesional corticosteroids are frequently touted as the first-line treatment for keloids, they are associated with a 1-year recurrence rate of 33%.^13^ Hypertrophic scars have for some time been treated with noninvasive techniques such as silicone gel sheets. However, these treatments are not without their own problems, requiring use for a prolonged period of time (many months usually) to obtain optimum results.^14^ The use of multimodal techniques combining surgical excision with other treatments such as steroids to treat keloids does improve outcomes, but can still have a recurrence rate of 11%–19%.^15^

This editorial has reflected upon the power of the gaze, its current ubiquitousness, and some of its consequences and challenges, in some cases augmented by the new—fleeting or permanent?—social norms brought about by the COVID-19 pandemic. The authors cannot change the emotions, judgements, or mores of society. However, they can seek to ameliorate the medical conditions noted above to diminish or remove the sometimes physically or emotionally devastating consequences of scarring. We cannot fight all of the “monster.” But to allude for the second time in this editorial to Nietzche's “monster”—perhaps that will save us from becoming monstrous ourselves…

“He who fights with monsters should look to it that he himself does not become a monster. And if you gaze long into an abyss, the abyss also gazes into you.”

—Friedrich Nietzsche
